# Antibody-mediated oral delivery of therapeutic DNA for type 2 diabetes mellitus

**DOI:** 10.1186/s40824-018-0129-7

**Published:** 2018-07-27

**Authors:** Seungbin Cha, Sun Hwa Lee, Sung Hun Kang, Mohammad Nazmul Hasan, Young Jun Kim, Sungpil Cho, Yong-Kyu Lee

**Affiliations:** 1 Department of Biomedical Chemistry, Konkuk University, Chungju, 27478 Republic of Korea; 2 KB-Biomed, Chungju, 27469 Republic of Korea; 3 Department of Chemical and Biological Engineering, Korea National University of Transportation, Chungju, 27469 Republic of Korea; 4 4D Biomaterials Center, Korea National University of Transportation, Jeungpyeong, 27909 Republic of Korea

**Keywords:** Type 2 diabetes, GLP-1, Oral gene delivery, FcRn

## Abstract

**Background:**

Diabetes mellitus (DM) is a chronic progressive metabolic disease that involves uncontrolled elevation of blood glucose levels. Among various therapeutic approaches, GLP-1 prevents type 2 diabetes mellitus (T2DM) patients from experiencing hyperglycemic episodes. However, the short half-life (< 5 min) and rapid clearance of GLP-1 often limits its therapeutic use. Here, we developed an oral GLP-1 gene delivery system to achieve an extended antidiabetic effect.

**Methods:**

Human IgG1 (hIgG1)-Fc-Arg/pDNA complexes were prepared by an electrostatic complexation of the expression plasmid with various ratios of the positively modified Fc fragments of an antibody (hIgG1-Fc-Arg) having a targeting ability to FcRn receptor. The shape and size of the complexes were examined by atomic force and field emission electron microscope. The stability of the complexes was tested in simulated gastrointestinal pH and physiological serum condition. Cellular uptake, transport, and toxicity of the complexes were tested in the Caco-2 cells. Biodistribution and antidiabetic effect of the complexes were observed in either Balb/c mice or Lep_db/db_ mice.

**Results:**

A 50/1 ratio of the hIgG1-Fc-Arg/pDNA produced a complex structure having approximately 40 ~ 60 nm size and also demonstrated protection of pDNA in the complex from the physiological pH and serum conditions. Cellular uptake and transport of the complex were demonstrated in Caco-2 cells having FcRn receptor expression and forming the monolayer-polarized structure. The cellular toxicity of both delivery vehicle and the complex revealed their minimal toxicity comparable with nontoxicity of a commercial transfection reagent. Biodistribution of the complex showed the detectable distribution of the complex in the most parts of gastrointestinal tract due to ubiquitous expression of the FcRn receptors. An in vivo type 2 diabetes treatment study of oral administration of hIgG1-Fc-9Arg/pGLP-1 complexes showed absorption and expression in GI tract of either Balb/c mice or Lep_db/db_ mice.

**Conclusion:**

In this study, we developed an oral GLP-1 gene delivery system on the platform of cationic hIgG1-Fc-9Arg. Prolonged t1/2, less immunoactivity, and better bioactivities of hIgG-Fc-9Arg/pGLP-1 complexes appeared to be a promising approach to achieve potent treatment of type 2 diabetes treatment.

**Electronic supplementary material:**

The online version of this article (10.1186/s40824-018-0129-7) contains supplementary material, which is available to authorized users.

## Background

Diabetes mellitus (DM), commonly known as diabetes, is a chronic, progressive metabolic disease of uncontrolled elevation of blood glucose levels [1]. According to a 2016 WHO report, the global burden of diabetes in 2014 was 422 million people, a prevalence of 8.5% among the total adult population [1]. Without proper management, complications including vision loss, kidney failure, cardiovascular disease, and lower limb amputation lead to premature death [1]. Type I diabetes (T1DM), characterized by the need for daily administration of insulin, results from autoimmune destruction of insulin-producing pancreatic β-cells [2, 3]. However, type II diabetes (T2DM), accounting for the majority of diabetes cases around the world, stems from either insulin resistance or insufficient insulin production [3, 4]. Even though there have been advances in the understanding of the pathogenesis and control of both forms of diabetes, therapeutic methods including insulin injection remain unsatisfactory [3]. Injectable insulin is not physiologically controlled and generates patient non-compliance, resulting in suboptimal control and ensuing diabetic complications. The glucose-lowing agents offered as alternatives to insulin injection include amylin analogs, sodium/glucose cotransporter-2 (SGLT-2) inhibitors and dipeptidyl peptidase IV (DPP-IV) inhibitors [5]. Hypoglycemia from these anti-diabetic agents is the significant adverse effect [5]. Among these glucose-lowering agents, glucagon-like peptide-1 (GLP-1) is a natural gut-derived small peptide hormone produced from intestinal L-cells after a meal [6]. GLP-1 has numerous roles as a physiological regulator of lowering hyperglycemic condition, and inhibition of gastric emptying and food intake [6]. The demonstrated antidiabetic effect of the GLP-1 has led to approval of the GLP-1 receptor agonist such as exendin-4 (Byetta®) that is strictly glucose-dependent, reducing the risk of hypoglycemia [5, 6]. While GLP-1 has its superior insulinotropic effect, use of GLP-1 for diabetes remains problematic because of its short plasma half-life (approximately 5 min) and rapid metabolic clearance [7–13].

Therapeutic gene delivery systems with various gene carriers including nonviral vectors are potential solutions to the limitations of current diabetic treatments. Nonviral gene delivery is achieved by either nano-size gene complexation or adsorption of the gene to nanoparticles through an interaction between negatively charged DNA and nonviral cationic carriers such as polymers, lipids and inorganic materials [8]. Recent advances and consideration of the prospects for their application provide better methods of diabetic treatment, primarily for the prevention and cure of T2DM. However, direct intravenous injection of these systems limited further application of gene delivery regarding serum stability, immunogenicity, low transfection efficiency and high costs, encouraging researchers to focus on alternative systems [8, 14–20].

Oral gene delivery is an attractive alternative to parenteral routes, offering several apparent advantages. First, ease of administration leads to improved patient convenience and compliance. Second, limited biodistribution by transfecting genes almost exclusively to intestinal epithelial cells is advantageous regarding safety concerns [21]. Furthermore, efficient oral gene delivery systems could lead to the expression and secretion of therapeutic proteins into the systemic circulation. Although proof of concept for oral gene therapy has already been provided, nonviral gene delivery through the gastrointestinal (GI) tract has rarely been investigated due to a reduced chance of protein expression by low gene transfection efficiency. Additionally, the GI tract itself provides barriers, leading to a reduction in the number of genes reaching a target in intact form. Most notably these barriers are low gastric pH, the mucus gel layer and enzymatic degradation caused by intestinal nucleases [21, 22].

Neonatal Fc receptor (FcRn) plays a role in transporting human IgG1(hIgG1) in breast milk from mother to offspring across the polarized intestinal layer. hIgG1 has a prolonged circulation time, with a half-life (t_1/2_) of 21 days compared with the t_1/2_ of other Ig classes. The complexation of hIgG1 to FcRn led to the enhancement of the t_1/2_ of hIgG1 and facilitated cellular transport across the intestinal layer [23–25]. The Fc portion of IgG binds with high affinity to FcRn in acidic pH (< 6.5) but not at a physiological pH (7.4). In the gut of neonatal rodents, after passing through the stomach, the slightly acidic stomach content containing maternal IgG pass into the duodenum. IgG binds to FcRn on the apical surface of epithelial cells. Following transcytosis process, FcRn releases bound IgG into the underlying extracellular space which is at physiological pH (7.4). In human, the syncytiotrophoblast internalizes fluid containing maternal IgG into endosomes, and it is gradually acidified, allowing IgG to bind tightly to FcRn present in the chamber. Then, the vesicle fuses with the membrane on the fetal side of the syncytiotrophoblast, where the dissociation of IgG happens in physiological pH [24]. The Fc fragment has been concerned with the prolonged survival of hIgG1 because Fc fragments had a similar t_1/2_ to that of intact hIgG1 and much longer survival than did Fab fragments [24]. This prolongation was interpreted by the existence of an Fc receptor preserving hIgG1 Fc fragments from normal lysosomal degradation [26, 27]. Discovery of the interaction of Fc of hIgG1 with FcRn provided an insight into the development of a carrier system to overcome GI barriers in oral gene delivery [23–25].

In this study, we developed an oral pβ-sp-GLP-1 gene delivery system on the platform of cationic hIgG1-Fc-9Arg. Bioactivity of this oral gene delivery system demonstrated the expression of a potential long-acting GLP-1 in T2DM db/db mice. Prolonged t_1/2_, less immunoactivity, and better bioactivities of hIgG-Fc-9Arg/pβ-sp-GLP-1 complexes suggested advantages over direct insulin injection or administration of chemical drugs for T2DM treatment.

## Methods

### Materials

Fluorescein isothiocyanate (FITC), bobo-3 iodide (570/602), and Lipofectamine™ Plus were purchased from Thermo Fisher Scientific (Waltham, MA, USA). Exfection™ LE Mini was purchased from GeneAll Biotechnology (Seoul, Korea). Minimum Essential Medium (MEM), RPMI 1640 and Dulbecco’s Modified Eagle’s Medium (DMEM), Dulbecco’s Phosphate Buffered Saline (PBS), and trypsin were purchased from Sigma Aldrich (Taufkirchen, Germany), and fetal bovine serum (FBS) was purchased from EMD Millipore (US Origin). Caco-2 and HT-29 (human epithelial colorectal adenocarcinoma cell lines), HEK-293 (human embryonic kidney cell line), and HeLa (human cervical carcinoma cell line) were purchased from the Korean Cell Line Bank (KCLB, Korea). The GLP-1 ELA kit was purchased from Sigma-Aldrich (St. Louis, MO, USA). Ultrasensitive mouse insulin ELISA kit was purchased from Morinaga (Yokohama, Japan). A pAcGFP-N1 expression plasmid was kindly provided by Dr. Young Jun Kim (Konkuk University, Chungju, Korea). A pβ-sp-GLP-1 expression vector was obtained from Dr. Minhyung Lee (Hanyang University, Seoul, Korea).

### Animals

Balb/c mice (5–7 weeks old) and Lep_db/db_ mice (male, 7–9 weeks old) were purchased from Daehan Bio Link, Inc. (Chungbuk, Korea). All mice were maintained in sterile, autoclaved cages, 3 per cage, on a standard chow diet. All animal experiments followed the guidelines established by Chonnam University Institutional Animal Care Use Committee and other proper approvals were obtained before the study.

### Preparation of hIgG1-Fc-9Arg

hIgG1-Fc-9Arg protein was prepared by a transient cell-based protein expression system. Briefly, the C-terminus of a hIgG1-Fc expression plasmid (Korea Research Institute of Bioscience & Biotechnology, South Korea) was extended with nine-arginine (9 Arg) sequences by PCR. The extension of 9 Arg from hIgG1-Fc was confirmed by both PCR amplification and DNA sequencing [28–31]. The hIgG1-Fc-9Arg expression plasmid was further introduced into Expi293F cells using ExpiFectamine™ 293 transfection reagent according to manufacturer’s instructions. At 6–7 days of cell culture after transfection, the cleared supernatants were harvested by centrifugation at 8000 rpm for 15 min and microfiltration with 0.22 μm microfilter. A-HiTrap Mabselect SuRe column (GE Lifesciences, Buckinghamshire, England) was used to separate the hIgG1-Fc-9Arg protein from the supernatants. The hIgG1-Fc-9Arg protein eluted from the column was neutralized with 1 M Tris (pH 8.5). After buffer exchange of the neutralized eluent with PBS containing 5% trehalose (pH 7.4), the hIgG1-Fc-9Arg protein solution was concentrated with ultracentrifugal filter (3 kDa Amicon Ultra 2 mL centrifugal filter, Millipore-Sigma, Burlington, MA, USA). The size of hIgG1-Fc-9Arg was confirmed by both SDS-PAGE and PAGE.

### Complexation of hIgG1-Fc-9Arg with pAcGFP-N1

Various weight ratios of hIgG1-Fc-9Arg /pAcGFP-N1 complexes were prepared in microcentrifuge tubes by addition of pAcGFP-N1 (1 μg/μl) into hIgG1-Fc-9Arg (9 μg/μl) following adjustment of final volume by PBS (pH 7.4) (Additional file 2). After stabilization of the complex with 30-min incubation at room temperature, the complex was applied to a DNA retardation assay to ensure DNA-complexation by electrophoresis on 1% agarose gels under tris-acetate-EDTA (TAE) buffer at 100 V for 30 min.

### Characterization of hIgG1-Fc-9Arg/ pAcGFP-N1 complex

The surface properties of hIgG1-Fc-9Arg/pAcGFP-N1 complexes (20/1, 50/1, 100/1, *w*/w) were analyzed by an atomic force microscope (AFM, Multimode-N3-AM, Bruker crop., Germany) in Tapping Mode™. The shape and size of an hIgG1-Fc-9Arg/ pAcGFP-N1 complex (50/1, w/w) were observed by a Field Emission Transmission Electron Microscope (FE-TEM, JEM-2100F, JEOL Ltd., Japan) at 20 kV in high vacuum mode [30]. The samples were prepared by spreading 1000-fold diluted hIgG1-Fc-9Arg/pAcGFP-N1 complexes on a copper grid and drying the samples.

### pH and serum stability of hIgG1-Fc-9Arg/pAcGFP-N1 complex

Orally administered biological compounds need to be stable without loss of their activity from pH-fluctuations during gastrointestinal transit and from serum instability during systemic circulation. To estimate the stability of hIgG1-FC-9Arg during gastrointestinal transit, hIgG1-FC-9Arg was incubated for 0, 30, and 60 min at various pH conditions. SDS-PAGE was performed to check the stability of hIgG1-FC-9Arg after incubation. The stability of hIgG1-Fc-9Arg/pAcGFP-N1 (50/1, *w*/w) complexes were also assessed by 1% agarose gel electrophoresis with samples that were prepared after incubation at various times at pH 2, the average pH of the stomach.

Various ratios of the complex were incubated for up to 24 h with 10% fetal bovine serum (FBS) at 37 °C to assess serum stability of the complexes. After incubation, samples were treated with EDTA to stop the further enzymatic reaction and were subjected to 1% agarose gel electrophoresis.

Samples were treated with 1% SDS to release plasmid DNA to estimate either pH or serum effect of the pAcGFP-N1 plasmid in the complex. Noncomplexed pAcGFP-N1 plasmid, a naked pDNA, was used as a comparison with the samples.

### FcRn-mediated cellular uptake of hIgG1-Fc-9Arg/pDNA complex

The expression of FcRn was measured by immunoblotting the cell extracts obtained from human epithelial colorectal adenocarcinoma cells (Caco-2, HT-29), human embryonic kidney cells (HEK 293), and human cervical carcinoma cells (HeLa). HEK293-FcRn cell extracts played the role of positive FcRn expression controls to ensure the expression of FcRN in the experimental samples.

Ten μg FITC-hIG1-Fc-9Arg conjugate was added to the HeLa, HEK293, and Caco2 cells for 1 h to check whether the FcRn cell surface receptor played a role in cellular uptake of the hIgG1-Fc-9Arg complex. The confocal microscopic analysis confirmed the green fluorescence emitted from FITC in the conjugate. In addition to FITC conjugates, 20:1, 50:1 and 100:1 ratio of the hIgG1-FC-9Arg/pAcGFP-N1 complex was added to the Caco-2 cells for 48 h, and GFP expression was monitored. CaCo-2 cells transfected with pAcGFP-N1 using lipofectamine were transfection controls to compare GFP expression from the experimental samples treated with the complex.

### Cellular toxicity of hIgG1-Fc-9Arg/pDNA complex

To determine the cellular toxicity of the complex, an MTT-based cell viability assay was performed in Caco-2 cells according to the manufacturer’s instructions. Caco-2 cells that were plated in the 96-well plates at 0.1 million cells per well at 24 h before the treatment were treated with the complex for 24 h. After treatment, culture media was replaced with MTT solution consisting of 20 μl MTT (5 mg/ml, *w*/*v*) solution and 180 μl media and were further incubated for 30 min to an hour at 37 °C to observe insoluble formazan crystals in the cells. After finishing MTT treatment, DMSO treatment completely solubilized the formazan crystals in the cells. The absorbance at 570 nm of the solution proportionally increased with the numbers of viable cells. The percentage cell viability was described by the following equation:

$$ \% viable\ cells=\frac{\left({abs}_{sample}-{abs}_{blank}\right)}{\left({abs}_{control}-{abs}_{blank}\right)}\times 100 $$

*abs*_*sample*_: absorbance of the sample with a treatment.

*abs*_*control*_: absorbance of the sample without treatment.

*abs*_*blank*_: absorbance of DMSO.

### Cellular transport of hIgG1-Fc-9Arg/pDNA complex across the Caco-2 cell monolayer

A transwell permeability assay with monolayered Caco-2 cells simulated cellular transport of materials from the apical region to basolateral region of the gastrointestinal tract [32–34]. Briefly, the complex prepared from various ratios of hIgG1-Fc-9Arg and bobo-3 pDNA was applied to the apical site of the Caco-2 cells monolayered on a transwell system (Corning® Transwell® polyester membrane pore size 0.4 μm, diameter 12 mm cell culture inserts, Millipore). The determination of apical-to-basolateral translocation of the complex was the measurement of fluorescence intensity emitted from the bobo-3 pDNA complex in the basolateral medium (HBSS, pH 7.4).

### Endosomal trafficking of hIgG1-Fc-9Arg complex

The experimental cells were the Caco-2 cell line for treatment of 10 μg FITC-hIgG1-Fc-9Arg complex for either 30 min or 4 h. The additional 10-min treatment of Caco-2 cells with lysotracker® led to staining late endosomes and lysosomes in the cells. The fluorescent images obtained from FITC and lysotracker in the cells indicated the endosomal locations of the complex.

Intracellular fluorescence emitted from the various ratios of hIgG1-Fc-9Arg/bobo-3 pDNA complex demonstrated the endosomal fate of the complexes. A positive control to monitor endosomal trafficking of the complexes was the cells treated with a commercial transfection reagent, Lipofectamine.

### Biodistribution of a FITC- hIGg1-Fc-9Arg complex

Balb/c mice (*n* = 3, male, 5–7 weeks) were the experimental animal given the oral administration of 10 μg FITC-hIgG1-Fc-9Arg complex. At either 1 or 3 h after oral feeding, various parts of organs from the mice were harvested and imaged to observe biodistribution of the complex by using a Kodak Digital Science™ Image Station 440CF (IS440CF) system. After imaging, the organs were further processed into a solution obtained from grinding the liquid nitrogen-frozen organ with mortar and pestle. Quantification of the organ distribution of the complex was performed by measurement of the fluorescence intensity emitted from FITC in the complex by microplate spectrometry.

### Antidiabetic effect of a hIgG1-Fc-9Arg/pGLP-1 complex

Lep_db/db (_*n* = 4, male, 7 weeks) and Balb/c (*n* = 3, male, 5–7 weeks) mice received oral administration of hIgG1-Fc-9Arg/ pβ-sp-GLP-1 complexes (50/1). The complex contained 20 μg of pβ-sp-GLP-1 and was given to the mice on 0, 7, 14, 21, 35, 44 days. Control mice received PBS by oral route following the same dosing schedule. At every oral dosing time, mice underwent measurement of glucose levels and body weight with a glucose meter and weight balance. Experimental mice supplied their blood from their abdominal-inferior vena cava after 8 h fasting for further processing of the serum by centrifugation. The performance of EIA (Sigma-Aldrich) and ELISA (Morinaga) with the serum provided information regarding the level of GLP-1 and insulin. HE histology analysis estimated intestinal tissue toxicity after oral administration of the complex to the mice.

### Statistical analysis

All applicable data are expressed as a mean ± standard deviation unless otherwise noted. Statistical analysis was performed with Prism software (version 7.04; GraphPad Software, La Jolla, CA, USA). Significance was set at *p* < 0.05.

## Results

### Preparation of hIgG1-Fc-9Arg protein

To develop a delivery system, we performed a PCR-modification of the hIgG1-Fc plasmid with nine-arginine sequences (9Arg-GGGs-9Arg) to produce a hIgG1-Fc-9Arg expression plasmid. Agarose gel electrophoresis indicated an amplified band with the size of approximately 850 bp originating from 9Arg sequences in the hIgG1-Fc-9Arg expression plasmid (Additional file 1). DNA sequencing further validated successful construction of the hIgG1-Fc-9Arg expression plasmid (Additional file 1). With this plasmid, Expi293F cells were transiently transfected to produce hIgG1-Fc-9Arg protein. Protein analysis revealed a successful generation of approximately 10 g/L fusion Fc protein. Additionally, the SDS-PAGE analysis of hIgG1-Fc-9Arg protein showed two separated bands with either 30 kDa or 35 kDa Additional file 1). Interestingly, native PAGE under nonreducing conditions revealed a 70 kDa single band (Additional file 1).

### Characterization of hIgG1-Fc-9Arg/pDNA complex

**Fig. 1:**
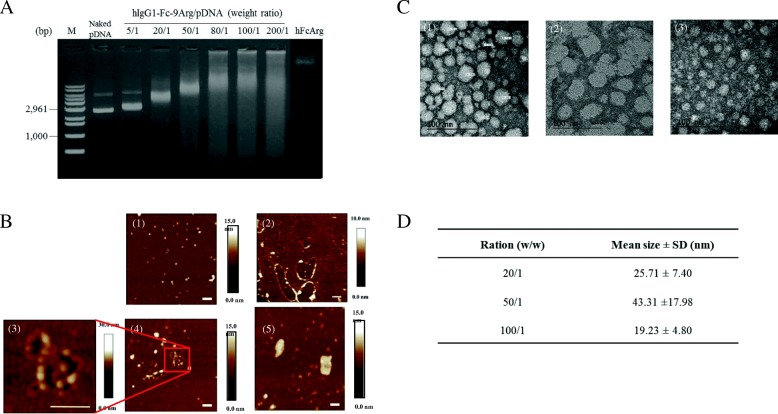
Characterization of hIgG1-Fc-9Arg/pDNA complex. **a** DNA retardation assay to confirm the complexation of hIgG1-Fc-9Arg with pAcGFP-N1. **b** AFM analysis to observe the surface structure of hIgG1-Fc-9Arg/pAcGFP-N1 complex in double distilled H_2_O (ddH_2_O) of pH 7.0. (1) hIgG1-Fc-9Arg only, (2) 20/1 weight ratio of hIgG1-Fc-9Arg/ pAcGFP-N1 complex, (3–4) 50/1 weight ratio of hIgG1-Fc-9Arg/pAcGFP-N1 complex, and (5) 100/1 weight ratio of hIgG1-Fc-9Arg/pAcGFP-N1 complex. Scale bar represents 200 nm. **c** FE-TEM analysis to observe the structure of hIgG1-Fc-9Arg/pAcGFP-N1 complex. The complex ratios are (a) (20/1, *w*/w), (b) (50/1, w/w), (c) (100/1 w/w). Scale bar indicates 100 nm. **d** The mean size of hIgG1-Fc-9Arg/ pAcGFP-N1 complex measured by FE-TEM. Mean ± S.D. from at least ten separate images of the complex

hIgG1-Fc-9Arg protein produced complexes with pDNA in various ratios by electrostatic interactions of positively charged hIgG1-Fc-9Arg protein with pDNA. (Additional file 2). Agarose gel electrophoresis showed a complexation of the protein with plasmid DNA (pDNA) by showing retardation of pDNA bands at the ratio from 20/1 up to 200/1 (Fig. 1a). Atomic force microscopy (AFM) exhibited surface properties of the complex of ratios of 20/1, 50/1, and 100/1 prepared in ddH_2_O of pH 7.0 (Fig. 1b (1)–(5)). Among these complexes, the 50/1 complex had a coiled DNA structure (Fig. 1b (3)–(4), suggesting a structure of the complex combined with proteins. Further analysis of the complex using FE-TEM revealed that the complex had a globular structure of nanometer size (Fig. 1c and d).

### pH and serum stability of hIgG1-Fc-9Arg/pDNA complex

**Fig. 2:**
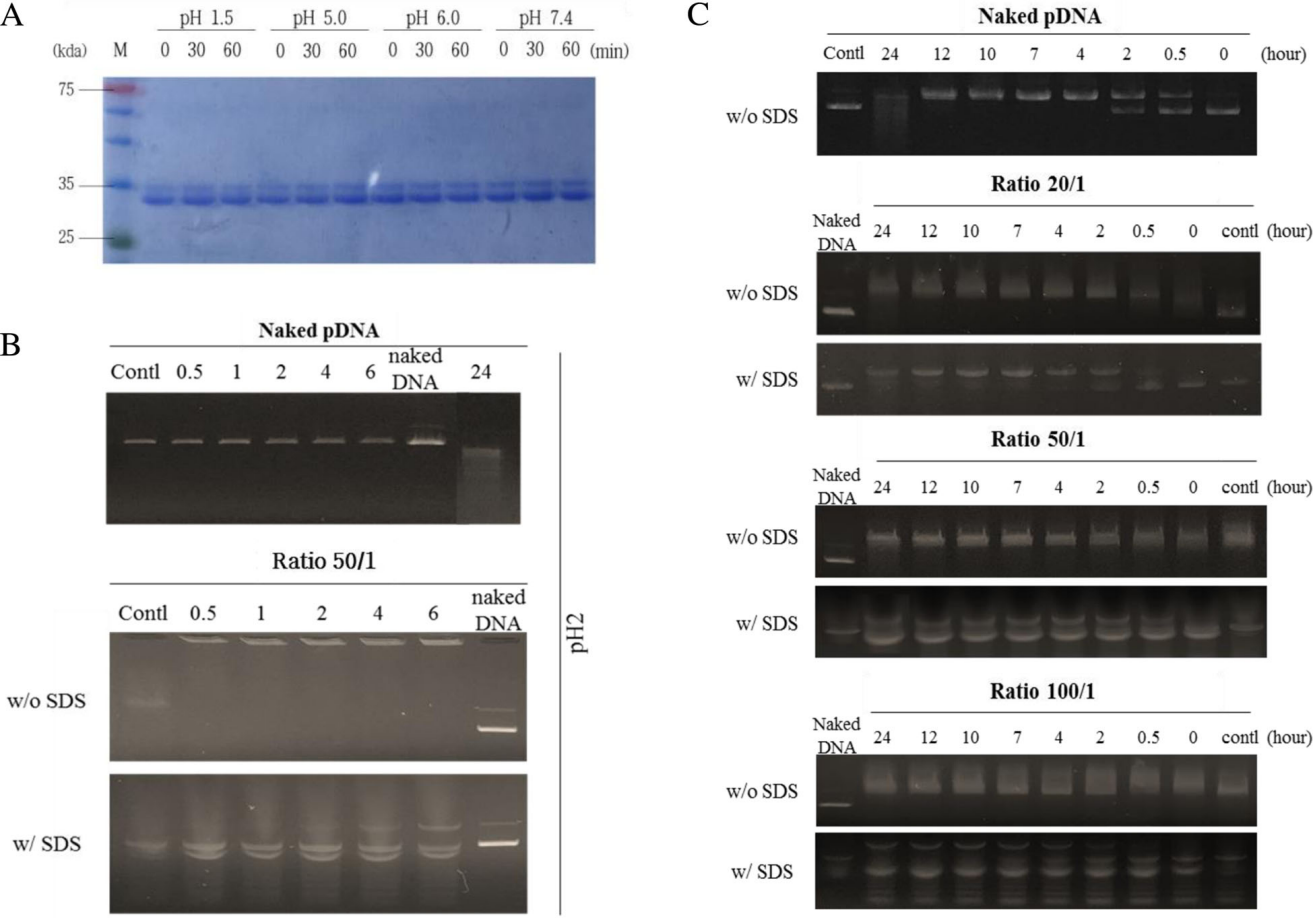
pH and serum stability of hIgG1-Fc-9Arg/pAcGFP-N1 complex. **a** Stability of the hIgG1-Fc-9Arg protein at various pH conditions (pH 1.5, 5.0, 6.0 and 7.4). **b** Stability of pDNA at 50/1 ratio of hIgG1-Fc-9Arg/pDNA complexes in the stomach environment (pH 2.0). **c** Serum stability of the complex. Naked pDNA and various ratios of hIgG1-Fc-9Arg/pDNA complexes were treated with 10% fetal bovine serum (FBS) at various time intervals (0, 0.5, 2, 4, 7, 10, 12, 24 h). Complexes were released by adding 1% SDS to determine the damage of pDNA. Representative images from at least three results

The measurement of pH effects on hIgG1-Fc-9Arg protein showed that the protein was stable under the range of pH values, mimicking gastrointestinal pH-fluctuations (Fig. 2a). The complex (50:1, pDNA: hIgG1-Fc-9Arg (*w*/w)) revealed maintenance of a complex structure over 6 h under stomach pH conditions (~ pH 2) without exposing the pDNA (Fig. 2b). The release of intact pDNA from the complex after SDS treatment further confirmed good protection of pDNA in the complex under acidic conditions (Fig. 2b).

The serum effects of the various ratios of the complex indicated that the complex maintained its stability with minimal release of pDNA under the 10% FBS condition for 24 h (Fig. 2c). However, the complex under FBS conditions released more pDNA as the complex had a higher pDNA ratio due to surpassing the complexation ability of the protein with pDNA.

### FcRn-mediated cellular uptake of hIgG1-Fc-9Arg/pDNA complex

**Fig. 3:**
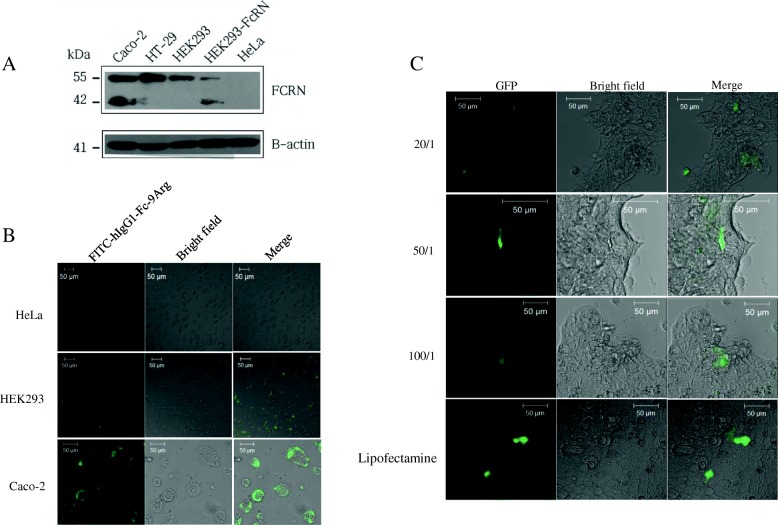
FcRn-mediated cellular uptake of hIgG1-Fc-9Arg/pDNA complex. **a** FcRn expression in human epithelial colorectal adenocarcinoma cell lines (Caco-2, HT-29), human embryonic kidney cell line (HEK 293), human cervical carcinoma cell line (HeLa), and HEK293-FcRn as a positive FcRn expression control. Representative image from immune- blots of total cellular protein. **b** Confocal microscopic images of HeLa, HEK293 and Caco-2 cells incubated with of 10 μg of FITC-hIgG1-Fc-9Arg for 1 h. **c** GFP expression in Caco-2 cells. Different ratios of hIgG1-Fc-9Arg/pAcGFP-N1 complexes were treated for 48 h. All cells were fixed after incubation with 4% PFA. Scale bar is 50 μm

Fc ligands in the hIgG1-Fc-9Arg/pDNA complex facilitate the binding of the complex to target cells that express FcRn receptors (Fig. 3a). The addition of FITC-hIgG1-Fc-9Arg complexes to Caco-2 and HEK 293 cells showed green fluorescent signals emitted from the FITC in the complexes (Fig. 3b). There was no FITC signal in the FcRn receptor-negative HeLa cells, indicating that the FITC signal came from the cellular uptake of the complex [23, 35]. Furthermore, stronger FITC signals from Caco-2 cells than from HEK 293 cells suggested that cellular uptake of the complex correlated with the level of FcRn receptor expression on the cell surface (Fig. 3b).

The treatment of a complex consisting of hIgG1-Fc-Arg with various ratios of pDNA (pGFP) led to the expression of GFP plasmid in Caco-2 cells (Fig. 3c). The over 50/1 ratio- complex showed better GFP expression than did the 20/1 ratio complex in the cells. The results suggested FcRn-mediated cellular uptake of the complex.

### Cellular toxicity of hIgG1-Fc-9Arg/pDNA complex

**Fig. 4:**
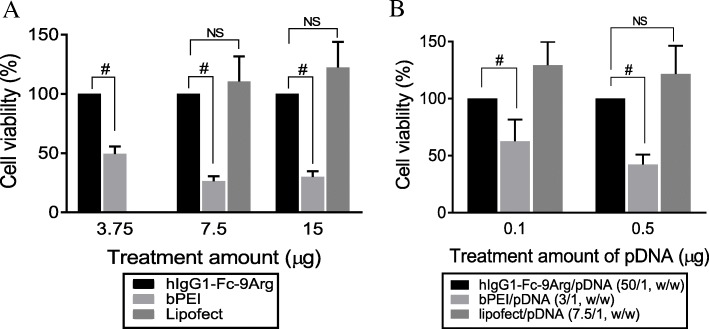
Relative cellular toxicity of hIgG1-Fc-9Arg/pDNA complexes. Caco-2 cells were incubated with hIgG1-Fc-9Arg/pDNA for 24 h. **a** MTT assay to estimate the cellular toxicity of hIgG1-Fc-9Arg protein to Caco-2 cells as a delivery vehicle. Mean ± SD (*n* = 5), 2-way ANOVA with Bonferroni’s multiple comparisons test. # was *P* < 0.05. **b** The estimation of the relative cellular toxicity of hIgG1-Fc-9Arg/pDNA complex evaluated by MTT assay. Relative cell viability indicated cellular toxicity of the complex. Relative cell viability obtained from the treatment with hIgG1-Fc-9Arg/pDNA complex was compared with that of cationic polymers (bPEI and lipofectamine). Mean ± SD (*n* = 5), 2-way ANOVA with Tukey’s multiple comparisons test. # was *P* < 0.05

The MTT-based assay in Caco-2 cells was performed to evaluate the potential cellular toxicity of both hIgG1-Fc-9Arg protein and its complex with pDNA. As a delivery vehicle of pDNA, hIgG1-Fc-9Arg protein showed almost 100% Caco-2 cell viability with the treatment of 3.75 μg, while bPEI maintained only 50% cell viability (Fig. 4a). At the comparisons of the cell viability with a high amount of delivery vehicles, hIgG1-Fc-9Arg demonstrated relatively minimal toxicity comparable with that of the nontoxicity of lipofectamine treatment in the cells (Fig. 4a). These experimental results suggested that hIgG1-Fc-9Arg protein is a safe delivery vehicle for pDNA to the cells.

The hIgG1-Fc-9Arg/pDNA complex (50/1, *w*/w) was safe for the survival of Caco-2 cells compared to the significant toxicity of the bPEI/pDNA complexes (Fig. 4b). Interestingly, the complex containing 0.5 μg pDNA, which is five times more amount than that of the complex containing 0.1 μg demonstrated the cellular safety of the complex similar to that of lipofectamine/pDNA complexes in Caco-2 cells (Fig. 4b).

### Cellular transport of hIgG1-Fc-9Arg complex across the Caco-2 cell monolayer

**Fig. 5:**
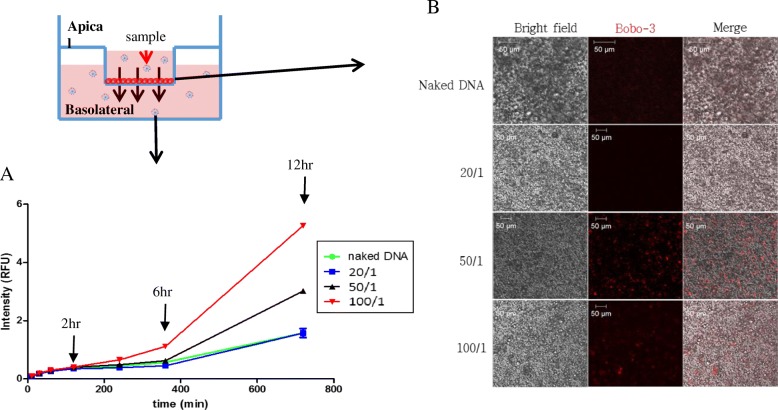
Cellular transport of hIgG1-Fc-9Arg/pDNA complexes across the Caco-2 cell monolayers. **a** Cell permeability assay in the Caco-2 monolayer to identify permeability of the complexes through the Caco-2 monolayer. Quantification of fluorescence intensity emitted from the bobo-3 pDNA in the complex indicated cellular transport of the complex from the apical to the basolateral site. **b** Cell images from confocal analysis of Caco-2 monolayers obtained after treatment of the hIgG1-Fc-9Arg/pDNA complexes. Cells were fixed with 4% PFA. Scale bar is 50 μm

Materials absorbed on the small intestine move from the apical to the basolateral region. Due to the formation of the monolayer-polarized structure, the Caco-2 cell line is a suitable experimental model for simulation of cellular transport of materials (Fig. 5a Top) [33]. The simulation of cellular transport of various ratios of hIgG1-Fc-9Arg complexes showed approximately 2 to 3 times more cellular transport in 50/1 and 100/1 complexes compared to that of naked pDNA, a negative experimental control (Fig. 5a Bottom).

Confocal microscopy analysis indicated movement of the complex from apical to basolateral regions, suggesting that the 50/1 ratio of the complex produced visible fluorescent images (Fig. 5b). Moreover, FITC-hIgG1-Fc-9Arg did not overlap with the late endosomes that were stained with lysotracker. However, the observation of the FITC signal in the endosomal complex at 4 h suggested the translocation of FITC-hIgG1-Fc-9Arg complex from the cell surface to the intracellular space through endosomal cycling (Additional file 3). Similarly, the addition of bobo-3 iodide-hIgG1-Fc-9Arg complex to Caco-2 cells showed strong red fluorescence signals in the cells. The 50/1 ratio of the complex had the best fluorescent signal compared to other ratios of the complex at intestinal pH (pH 6.0) (Additional file 3). These observations further confirmed the intracellular movement and expression of the pDNA combined with hIgG1-Fc-9Arg.

### Biodistribution and antidiabetic effect of a hIGg1-Fc-9Arg complex

**Fig. 6:**
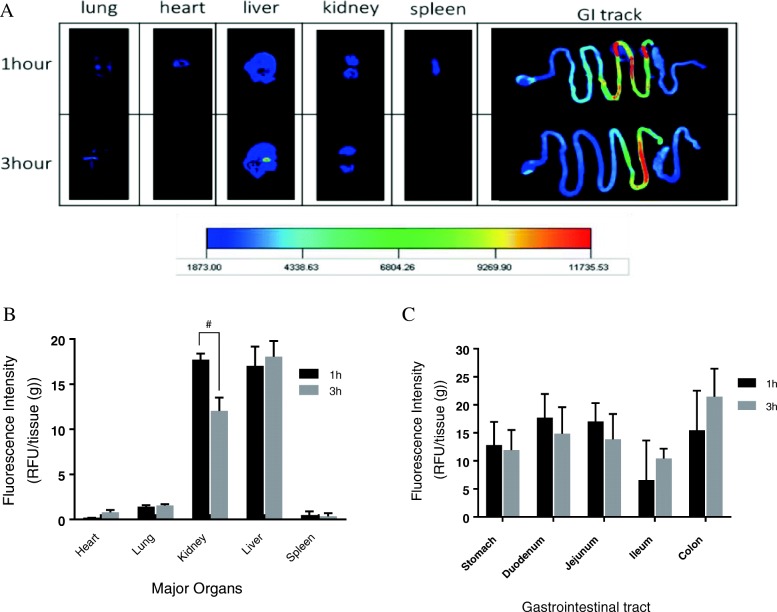
Distribution of the FITC-hIgG1-Fc-9Arg complexes to organs and gastrointestinal (GI) track in Balb/c mice (*n* = 3, male, 5–7 weeks). **a** Fluorescence images of FITC-hIgG1-Fc-9Arg in the major organs and GI tract. **b**-**c** Quantification of the fluorescence intensity is indicating biodistribution of the complexes. Mean ± SD (*n* = 2 or 3), 2-way ANOVA with Sidak’s multiple comparisons test. # was *P* < 0.05

Oral administration of the FITC-hIgG1-Fc-9Arg complex (1:50 ratio, *w*/w) to the mouse indicated explicit organ distribution of the complex by emitting fluorescence signals at either 1 or 3 h’ post-administration (Fig. 6a). Fluorescence signals from the primary organ and GI tract in Balb/c mice demonstrated evidence of signals mostly from the major organs, including liver and kidney, as well as some parts of the GI tract, including the small intestine (Fig. 6a). Quantification of the fluorescence signals indicated stronger signals from liver and kidney than from other organs (Fig. 6b). Interestingly, detectable signals from the GI tract including stomach, small intestine, and colon suggested the ubiquitous distribution of FcRn receptors in the GI tract with emphasis on slightly more distribution in the upper parts of the small intestine and colon (Fig. 6c).

In the in vivo study, leptin receptor-deficient mice treated with the GLP-1 gene developed mild hyperglycemia for eight weeks. The plasma levels of active GLP-1 in the hIgG1-Fc-9Arg/pβ-sp-GLP1 group increased nearly five-fold compared with the control groups in db/db mice (Additional file 4). However, serum insulin levels, blood glucose levels, and body weights were not significantly different but only slightly increased or decreased between control and oral administration mouse models (Additional file 4). In addition, H&E histology of the jejunum revealed negligible cellular toxicity and inflammation from oral administration of hIgG1-Fc-9Arg and gene complexes (Additional file 4).

## Discussion

Glucagon-like peptide-1 (GLP-1) is an antidiabetic hormone released from intestinal L cells into the circulation system. GLP-1 prevents type 2 diabetes mellitus (T2DM) patients from hyperglycemic episodes [20]. However, short biological half-life (< 5 min) and rapid clearance of GLP-1 often limit its therapeutic usefulness. Here, we developed a GLP-1 gene delivery system to achieve the extended antidiabetic effect by delivery of a GLP-1 expression plasmid (Additional file 5). The GLP-1 gene delivery system was a nano-complex consisting of GLP-1 expression plasmid and its delivery vehicle, a positively modified Fc fragment (hIgG1-Fc-Arg) having targeting ability for FcRn receptor located on intestinal L cells [24].

Design of the delivery vehicle is based on the utilization of interaction between hIgG1-Fc and FcRn receptor on intestinal L cells, which are predominantly located in the ileum and colon as open-type epithelial cells directly interacting with nutrients in the intestinal lumen [6]. In addition, hIgG isotypes had better affinities (i.e., lower K_D_) for mFcRn than did hFcRn [36]. For the delivery of negatively charged plasmids, modification of hIgG1-Fc was performed by adding nine arginine sequences to confer positive charges to hIgG1-Fc [35–37]. During the confirmation of a protein structure of hIgG1-Fc-Arg, which was produced from in vitro translation, native PAGE revealed a single band under nonreducing condition while two separated bands were observed in SDS-PAGE under reducing condition. These results imply that a bivalent antibody-like structure of hIgG1-Fc has not affected by the extension of 9 Arg through the genetic modification process. From the complexation of hIgG1-Fc-Arg with pDNA, we could detect size information from the FE-TEM analysis. However, we could not detect particle size or surface charge with conventional analytical tools such as dynamic light scattering (DLS) or zeta potential (ZP). We believe that the structural instability from the weak electrostatic interaction between pDNA and protein, including only 9 Arg positive charges, could limit the robust application of the DLS and ZP to measure both size and charge of the complexes [38–41].

For the development of an oral delivery system with nanocarriers through electrostatic interaction, the effect of fluctuations in pH during gastrointestinal transit and serum in the systemic circulation should be considered as biochemical barriers [39, 40]. Our simulated results under the physiological condition of pH fluctuations and serum concentration indicated that complex having over 50/1 ratio did not lose its stability and provided protection of its pDNA. Considering isoelectric point (pI) of 10.8 in arginine, we believe intestinal pH changes ranged from pH 2 to 8 did not change electrical charges in hIgG1-Fc-Arg, therefore; electrostatic complexation with pDNA seemed to maintain the complex under pH fluctuations.

The cellular uptake of the complex under a simulated intestinal condition presented with the polarized-monolayer Caco-2 cell system [25, 33] demonstrated uptake of the complex through an interaction between Fc ligand and FcRn receptor existed on the surface of Caco-2 cell membrane [23, 35]. Furthermore, cellular transportation of the complex suggested that the complex could have the dual function of both facilitation of cellular uptake and cellular transportation. Beside these abilities of the complex, we could confirm that the complex has minimal cellular toxicity comparable with nontoxicity of a commercial transfection reagent. Considering oral administration of the hIgG1-Fc-9Arg/pDNA complexes, we could insist safety of current nano-complex over other cation based-complex systems [38, 42].

The biodistribution of the nano-complex in an animal model demonstrated detectable distributions in the most parts of major organs and gastrointestinal tract. The gradual increase of biodistribution of the complex in the ileum and colon over time is also matched with the previous report about the predominant distribution of FcRn receptors on the surface of ileum and colon [6]. An in vivo type 2 diabetes treatment study showed the enhancement of serum GLP-1 from oral administration of a hIgG1-Fc-9Arg/pGLP-1 complex to the leptin-receptor deficient mouse (Lep_db/db_). However, body weight and blood glucose level seemed to show marginal changes. We assume that extreme hyperglycemic condition reaching to ~ 600 mg/dl of blood glucose level in lep_db/db_ mouse compared to an average of ~ 200 mg/dl of that in human T2DM patients led to masking the noticeable regulatory effect of GLP-1 [43, 44]. With this limitation of the current study, we believe that efficacy of the current nano-complex containing GLP-1 can be warranted in using better diabetic mouse model having the similar hyperglycemic condition to human T2DM. Lastly, it is noteworthy to mention the nano-complex did not show intestinal toxicity, especially jejunal tissue known as FcRn rich cells targeted by current hIgG1-Fc-9Arg/pDNA complex system.

## Conclusion

In conclusion, we have demonstrated that oral gene delivery with a hIgG1-Fc-9Arg gene carrier targeted FcRn receptor and facilitated transepithelial transport of the gene complex. Oral delivery of pβ-sp-GLP-1 gene with hIgG1-Fc-9Arg in type 2 diabetic animal model revealed stimulation of GLP-1 expression, suggesting a promising approach for the treatment of type 2 diabetes.

## Additional files

Additional file 1: Preparation of hIgG1-Fc-9Arg. **A**. C-terminal 9Arg extension of human IgG1-Fc by PCR. Lane 1. hIgG1-Fc-9Arg PCR (annealing Tm:55, 850 bp), Mw. DNA ladder, lane 2. hIgG1-Fc-9Arg PCR (annealing Tm:52, 850 bp). Template for PCR was an Avastin heavy chain. **B**. DNA sequence analysis of hIgG1-Fc-9Arg expression vector. **C**. SDS-PAGE with reduced hIgG1-Fc-9Arg. Mw. protein ladder (10–245 kDa), lane1. 0.1 μg, lane 2. one μg, land 3. ten μg and PAGE with non-reduced hIgG1-Fc-9Arg (lane 1) and Mw (protein ladder (10–245 kDa)). (PPTX 314 kb)

Additional file 2: Complexation of hIgG1-Fc-9Arg with pAcGFP-N1. (PPTX 36 kb)

Additional file 3: Endosomal trafficking of hIgG1-Fc-9Arg complex **A**. Cellular uptake and endosomal escape of FITC-hIgG1-Fc-9Arg in Caco-2 cells. **B**. Determination of bobo-3-pDNA complexed with hIgG1-Fc-9Arg on 50/1 weight ratio for tracking complexes mediated FcRn and evaluation of presence pDNA in Caco-2 cells. Lipofectamine used as positive control. Scale bar is 50 μm. (PPTX 1263 kb)

Additional file 4: Anti-diabetic effect and tissue toxicity of the complex. **A** Anti-diabetic effect of orally administered hIgG1-Fc-9Arg/pGLP-1 complex (20/1) and H&E histology analysis in balb/c mice (*n* = 3, male, 5–7 weeks) as a normal mouse and lep_db/db_ mice (*n* = 4, male, seven weeks) as a db/db mouse. (a) Serum insulin concentration, (b) serum GLP-1 concentration, (c) body weight, (d) blood glucose level at day 44 after continuous oral administration of the complex and its comparison of with the blood glucose level before administration of the complex (day 0). **B** H&E histology analysis of jejunum tissue from the small intestine after oral administration of the complex. (PPTX 1853 kb)

Additional file 5: The strategy of the intestinal receptor-mediated delivery of therapeutic gene. (PPTX 547 kb)
