# Supplementary figures and images for: Antibody-mediated oral delivery of therapeutic DNA for type 2 diabetes mellitus
Source: Biomater Res. 2018 Jul 27;22:19. doi: 10.1186/s40824-018-0129-7 (PMC6062860; doi:10.1186/s40824-018-0129-7)

## Slide 1
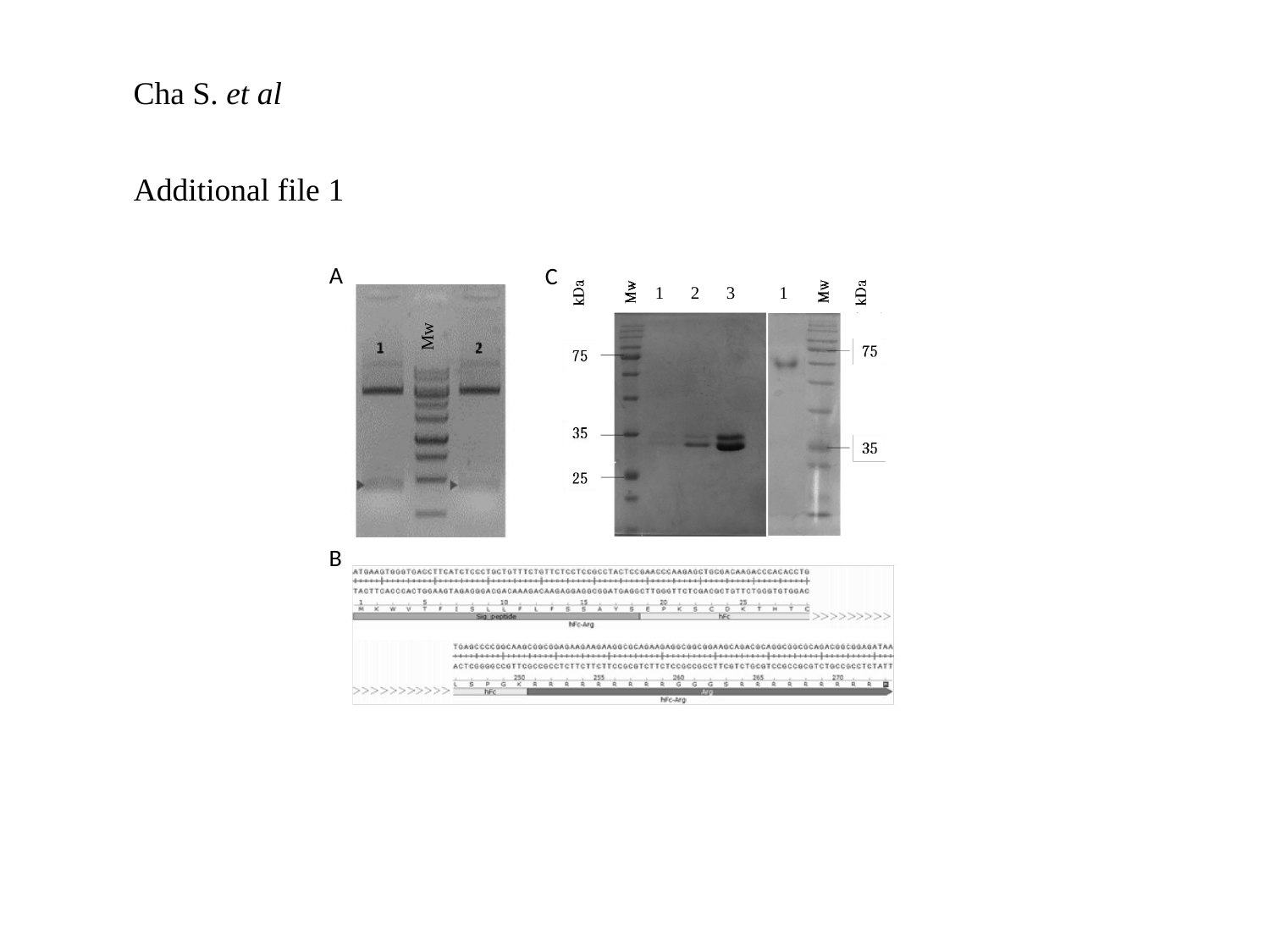

Cha S. et al
Additional file 1
A
C
1
1 2 3
Mw
B

Supplement: Supplementary file 1 — Preparation of hIgG1-Fc-9Arg. A. C-terminal 9Arg extension of human IgG1-Fc by PCR. Lane 1. hIgG1-Fc-9Arg PCR (annealing Tm:55, 850 bp), Mw. DNA ladder, lane 2. hIgG1-Fc-9Arg PCR (annealing Tm:52, 850 bp). Template for PCR was an Avastin heavy chain. B. DNA sequence analysis of hIgG1-Fc-9Arg expression vector. C. SDS-PAGE with reduced hIgG1-Fc-9Arg. Mw. protein ladder (10–245 kDa), lane1. 0.1 μg, lane 2. one μg, land 3. ten μg and PAGE with non-reduced hIgG1-Fc-9Arg (lane 1) and Mw (protein ladder (10–245 kDa)). (PPTX 314 kb) [file 40824_2018_129_MOESM1_ESM.pptx]

## Slide 1
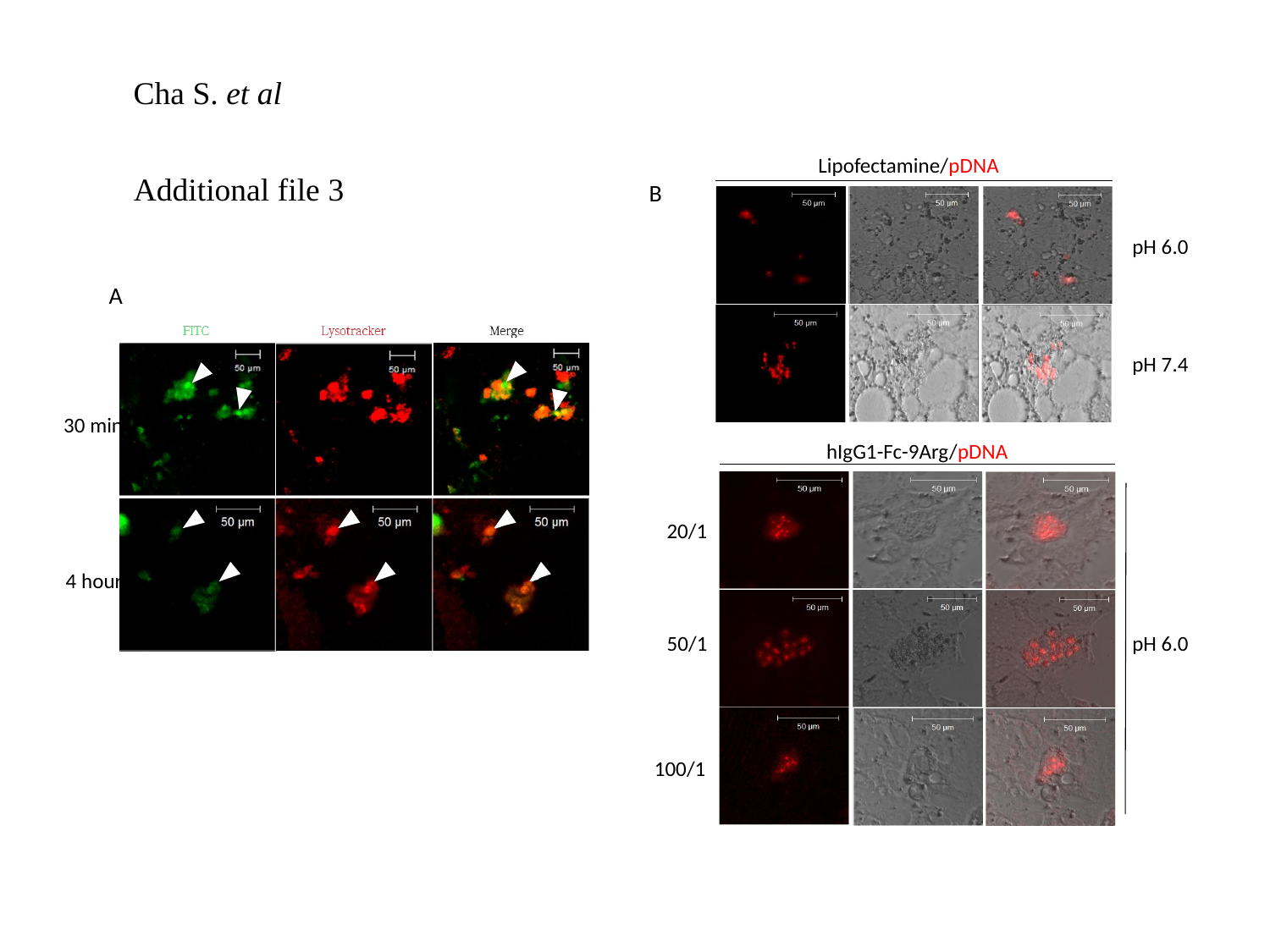

Cha S. et al
Lipofectamine/pDNA
pH 6.0
pH 7.4
hIgG1-Fc-9Arg/pDNA
20/1
50/1
pH 6.0
100/1
Additional file 3
B
A
30 min
4 hour

Supplement: Supplementary file 3 — Endosomal trafficking of hIgG1-Fc-9Arg complex A. Cellular uptake and endosomal escape of FITC-hIgG1-Fc-9Arg in Caco-2 cells. B. Determination of bobo-3-pDNA complexed with hIgG1-Fc-9Arg on 50/1 weight ratio for tracking complexes mediated FcRn and evaluation of presence pDNA in Caco-2 cells. Lipofectamine used as positive control. Scale bar is 50 μm. (PPTX 1263 kb) [file 40824_2018_129_MOESM3_ESM.pptx]

## Slide 1
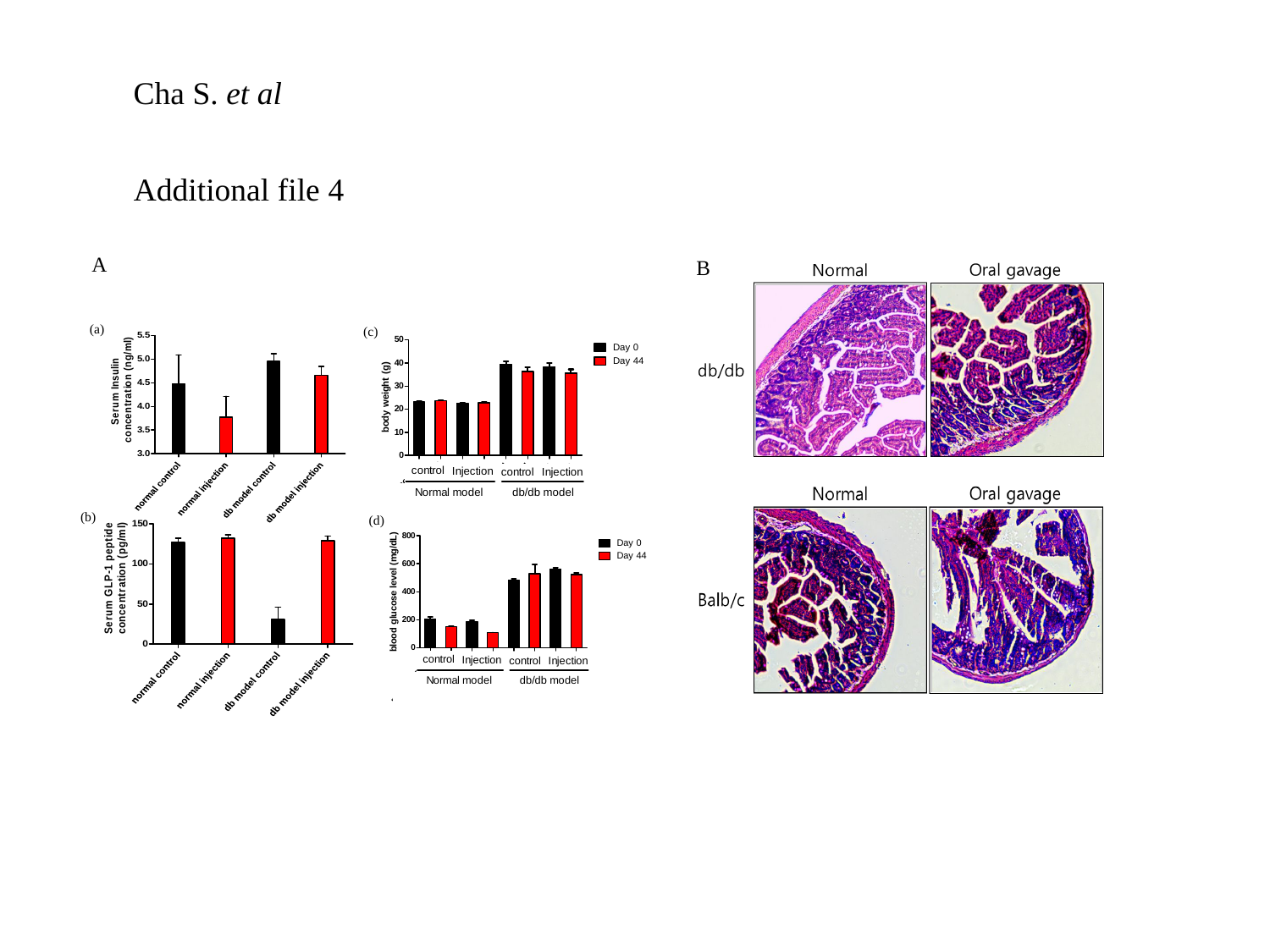

Cha S. et al
Additional file 4
A
B
(a)
(c)
(b)
(d)

Supplement: Supplementary file 4 — Anti-diabetic effect and tissue toxicity of the complex. A Anti-diabetic effect of orally administered hIgG1-Fc-9Arg/pGLP-1 complex (20/1) and H&E histology analysis in balb/c mice (n = 3, male, 5–7 weeks) as a normal mouse and lepdb/db mice (n = 4, male, seven weeks) as a db/db mouse. (a) Serum insulin concentration, (b) serum GLP-1 concentration, (c) body weight, (d) blood glucose level at day 44 after continuous oral administration of the complex and its comparison of with the blood glucose level before administration of the complex (day 0). B H&E histology analysis of jejunum tissue from the small intestine after oral administration of the complex. (PPTX 1853 kb) [file 40824_2018_129_MOESM4_ESM.pptx]

## Slide 1
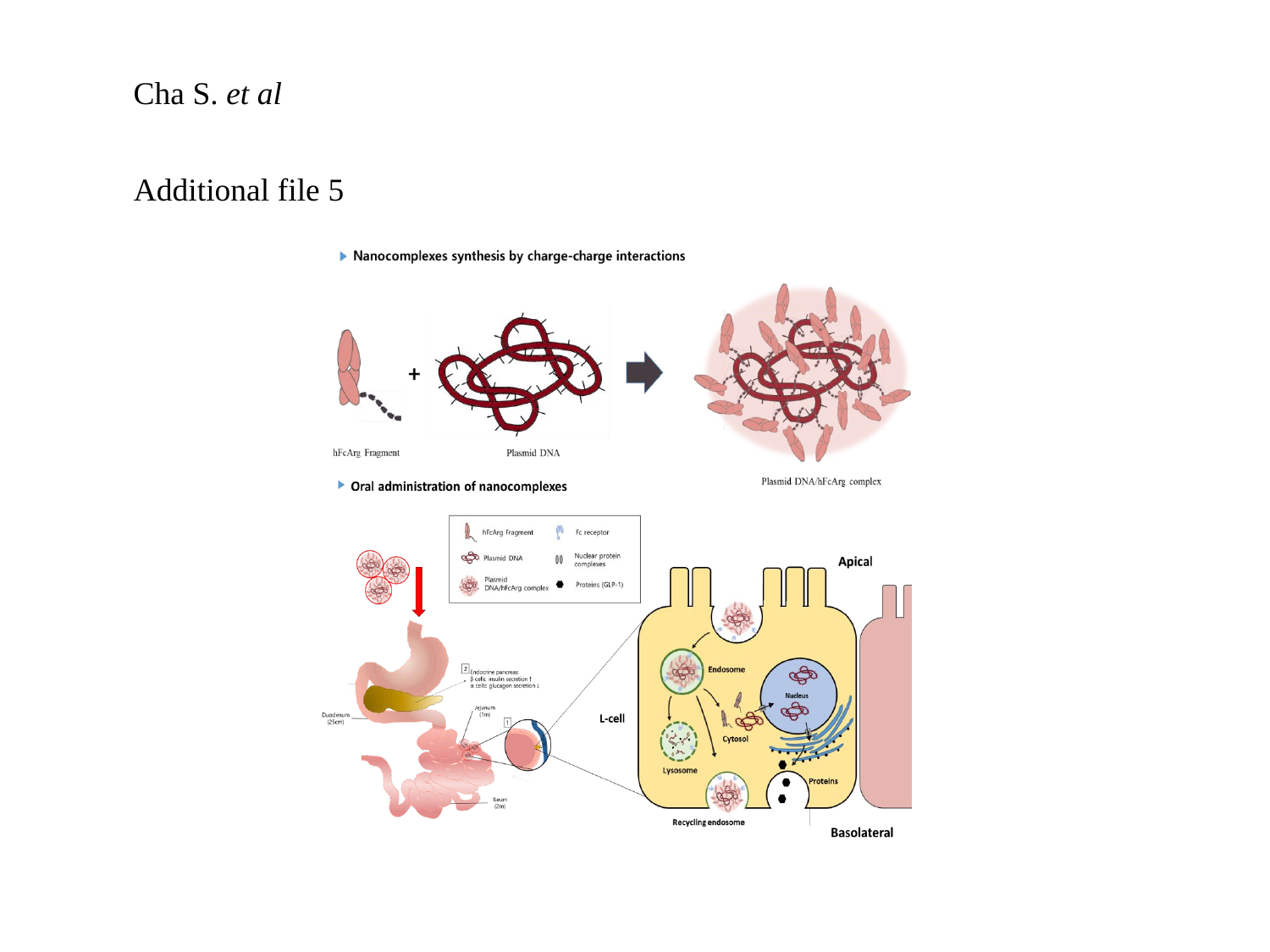

Cha S. et al
Additional file 5

Supplement: Supplementary file 5 — The strategy of the intestinal receptor-mediated delivery of therapeutic gene. (PPTX 547 kb) [file 40824_2018_129_MOESM5_ESM.pptx]
