# Supplementary material for: Antibody-mediated oral delivery of therapeutic DNA for type 2 diabetes mellitus
Source: Biomater Res. 2018 Jul 27;22:19. doi: 10.1186/s40824-018-0129-7 (PMC6062860; doi:10.1186/s40824-018-0129-7)
Supplement: Supplementary file 2 — Complexation of hIgG1-Fc-9Arg with pAcGFP-N1. (PPTX 36 kb) [file 40824_2018_129_MOESM2_ESM.pptx]

## Slide 1
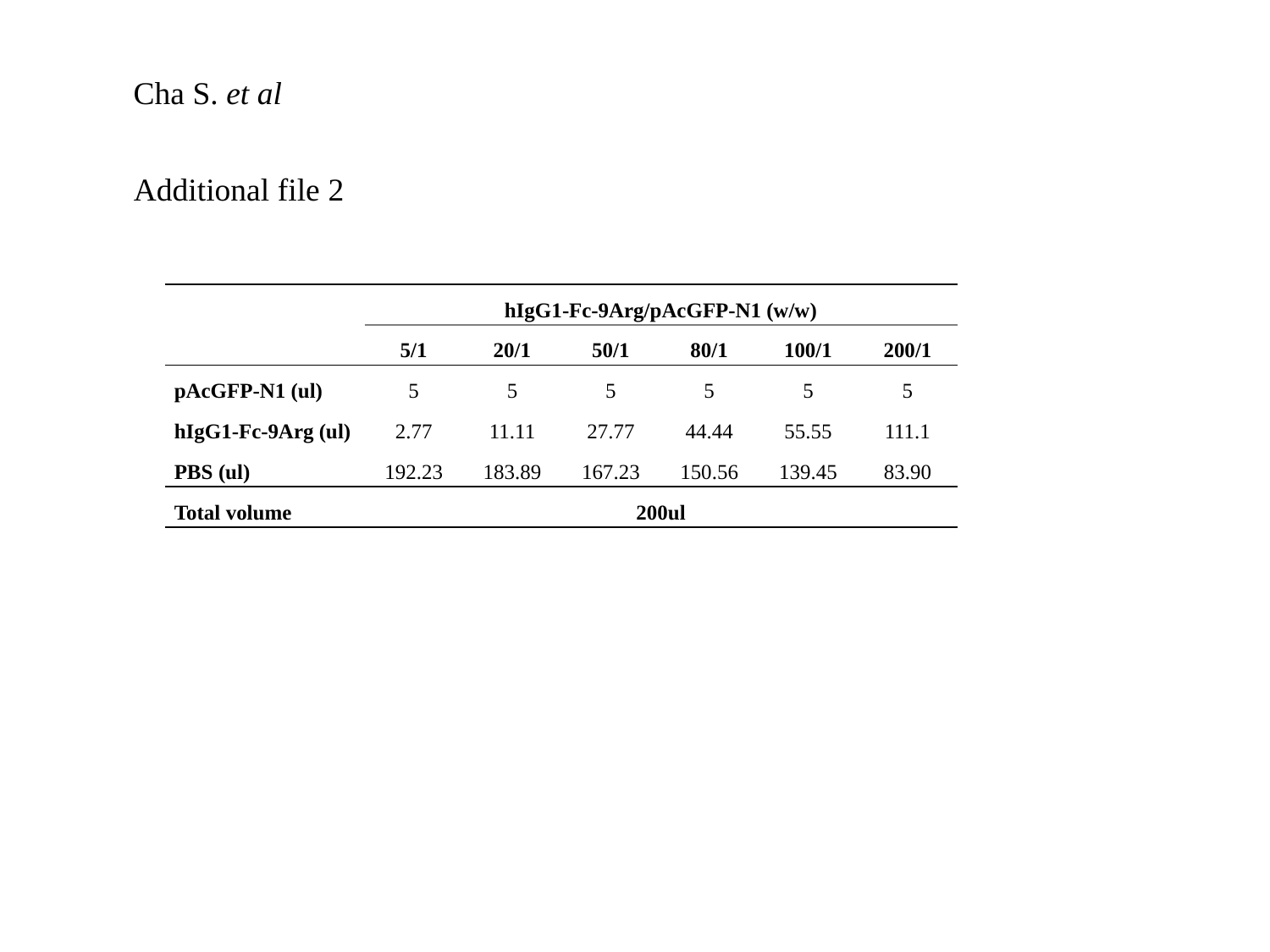

Cha S. et al
Additional file 2
| | hIgG1-Fc-9Arg/pAcGFP-N1 (w/w) | | | | | |
| --- | --- | --- | --- | --- | --- | --- |
| | 5/1 | 20/1 | 50/1 | 80/1 | 100/1 | 200/1 |
| pAcGFP-N1 (ul) | 5 | 5 | 5 | 5 | 5 | 5 |
| hIgG1-Fc-9Arg (ul) | 2.77 | 11.11 | 27.77 | 44.44 | 55.55 | 111.1 |
| PBS (ul) | 192.23 | 183.89 | 167.23 | 150.56 | 139.45 | 83.90 |
| Total volume | 200ul | | | | | |
